# Emoji as Affective Symbols: Affective Judgments of Emoji, Emoticons, and Human Faces Varying in Emotional Content

**DOI:** 10.3389/fpsyg.2021.645173

**Published:** 2021-04-20

**Authors:** Brigitte Fischer, Cornelia Herbert

**Affiliations:** Applied Emotion and Motivation Psychology, Institute of Psychology and Education, Ulm University, Ulm, Germany

**Keywords:** affective rating, emoji, emoticon, faces, valence, arousal, emotionality

## Abstract

An important function of emoji as communicative symbols is to convey emotional content from sender to receiver in computer-mediated communication, e. g., WhatsApp. However, compared with real faces, pictures or words, many emoji are ambiguous because they do not symbolize a discrete emotion or feeling state. Thus, their meaning relies on the context of the message in which they are embedded. Previous studies investigated affective judgments of pictures, faces, and words suggesting that these stimuli show a typical distribution along the big two emotion dimensions of valence and arousal. Also, emoji and emoticons have been investigated recently for their affective significance. The present study extends previous research by investigating affective ratings of emoji, emoticons and human faces and by direct comparison between them. In total, 60 stimuli have been rated by 83 participants (eight males, age: 18–49 years), using the non-verbal Self-Assessment Manikin Scales for valence and arousal. The emotionality of the stimuli was measured on a 9-point Likert scale. The results show significant main effects of the factors “stimulus category” and “discrete emotion” including emotionality, valence and arousal. Also, the interaction between these two main factors was significant. Emoji elicited highest arousal, whereas stimuli related to happiness were rated highest in valence across stimulus categories. Angry emoji were rated highest in emotionality. Also, the discrete emotion was best recognized in emoji, followed by human face stimuli and lastly emoticons.

## Introduction

In daily life and especially in face-to-face (F2F) communication, humans are able to express their feelings and states by changing their emotional facial expressions. Nevertheless, these facial expressions are not only demonstrating feelings, but also other information, including mood and can be accompanied by gestures, prosody and contextual cues (Aldunate and González-Ibáñez, 2017). Nowadays, people are confronted with a lot of social exchange situations which are not comparable to F2F-communication, for example in computer or mobile-based communication (CMC) platforms. Via Email, Instant Messaging Chats (e.g., Facebook) or social media platforms (e.g., Instagram) the written communication has enormously grown. Within this type of communication emoji or emoticons are available to still be able to express our feelings to our chatting partners.

The word emoticon is based on the words “emotion icon” (Pavalanathan and Eisenstein, 2015). Emoticons are also called typographic or text-based emoticons because they consist of typographic ASC-II character symbols (Huang et al., 2008; Guibon et al., 2016). Regarding emoticons, we differentiate between western style emoticon, as e.g.,:-), and eastern style emoticons: ^∧^_^∧^ (Rodrigues et al., 2018). Later on, emoji were designed by Shegetaka Kurita. The main difference between emoji and emoticons is that emoji are not rotated and presented in colors (Ganster et al., 2012). Today, emoji illustrate much more than only facial expressions, feelings or emotions, but also abstract concepts, hand gestures, animals, plants, objects or activities (Rodrigues et al., 2018). At the moment 3.304 emoji exist to express oneself emotionally (Emojipedia, 2020).

Keeping this in mind, emoji are not only nice decorative symbols in text messages but obtain several functions. According to Kaye et al. (2016) two key functions are to display emotional as well as social meanings and to reduce the ambiguity of the message. Further functions of emoji and emoticons are the providing of contextualization cues, such as markers of positive or negative attitudes, as well as the organizational role of social relationships. This means that interpersonal space can be reduced through reducing impersonality (Skovholt et al., 2014). Additionally, emoji and emoticons are often used because people think they are fun (Guibon et al., 2016) as well as to loosen up conversation (Adrianson, 2001).

Emoji and emoticons can be ambiguous because no concrete labels exist for them. Furthermore, the meaning of an emoji or emoticon relies often on the context of a message and they are learned intuitively. That is why it is hard to interpret them correctly which can lead to misunderstandings. Nowadays, not only the iPhone uses emoji but also other mobile phone devices. That is why emoji can vary in their representation dependent on the mobile phone system (iOS or Android) or social platform (Facebook or Twitter) you use. On Emojipedia, a website listing all different emoji variants, they mention at least 17 different representation forms of emoji due to platforms (Miller et al., 2016). As a consequence, an emoji sent by one person can generate a completely different emotion for the recipient, just because of using different devices. However, it should be mentioned that these presentation differences occur only for emoji but not for emoticons, as they are based on the ASC-II character sequences (Miller et al., 2016). Due to the fact that users interpret emoji or emoticons differently from what they—according to Unicode—actually mean, one aim of this study was to achieve ratings of the emotional meanings of faces, emoji and emoticons.

Emoji and emoticons are emotional stimuli highly varying in emotional content. Therefore, great differences on affective dimensions (as e.g., valence and arousal) could be expected. To evoke positive or negative emotions, stimulus material, as the International Affective Picture System (IAPS, Lang et al., 2008), the International Affective Digitized Sounds (IADS-2, Bradley and Lang, 2007), films (Ekman et al., 1987) as well as verbal attacks, insults and apologies (Atkinson and Polivy, 1976, Rodriguez Mosquera et al., 2000) have been used, measured by the Self-Assessment Manikin Scale (SAM, Bradley and Lang, 1994). The SAM-scale represents a non-verbal and culture-free pictoral instrument for assessing affective reactions (Bradley and Lang, 1994). Inducing emotions in a verbal or written manner can also be achieved simply by utilizing emotional words, compared to e.g., neutral words. Bradley and Lang already showed in 1999 that a correlation between valence and arousal ratings (affective space) exists in English words. Redondo et al. (2007) could also show this typical boomerang curve by adapting the English word database ANEW (affective norms for English words; Bradley and Lang, 1999) for Spanish words. Soares et al. (2012) could replicate these finding for Portuguese words as well. Finally, also a German word database exists, namely “The Berlin Affective Word List Reloaded” (BAWL-R). Their results showed also the typical quadratic function of relation between valence and arousal in the affective space (Vo et al., 2009).

A research study from Rodrigues et al. (2018) aimed to generate an emoji and emoticon database, which includes ratings on different dimensions (valence, arousal, familiarity, aesthetic appeal, visual complexity, clarity, and meaningfulness). Their data showed that emoji were rated more positive and more arousing than emoticons. Moreover, emoji achieved higher rates on the aesthetical appeal dimension, familiarity, clarity, and meaningfulness. Regarding the valence and arousal findings, they showed that emoticons were distinguished in positive, negative and neutral valence categories, whereas emoji only differed in the negative and positive valence category. Emoji also showed higher arousal scores than emoticons, which had a moderate arousal level. There were no significant correlations between valence and arousal dimensions, but a significant correlation between valence and meaningfulness as well as arousal and meaningfulness. An examination from Novak et al. (2015) on the sentiment of emoji classified emoji into positive, negative and neutral categories. Their results showed that the majority of negative emoji are sad faces, whereas positive emoji did not only consist of happy faces, but also of symbols (e.g., heart, party) as well as objects (wrapped gift, trophy). Regarding the neutral emoji, they found ambiguous results. The neutral condition contained signs as the Ying-and-Yang-emoji but also facial emoji with a rather negative connotation (e.g., face with cold sweat or crying face). Due to these results, an investigation from Shoeb and de Melo (2020) examined the association between emoji and specific emotions. Their results showed that only a few emoji show a relation to emotions as anger, disgust, fear, sadness or surprise. In contrast, the emotion joy reveals a wide range of associations from face emoji to object-emoji and concept-emoji.

In contrast to emoji, human faces are unique in terms of communication, because they can be very specific but also universal (Ekman and Friesen, 1982; Aldunate and González-Ibáñez, 2017). Ekman and Friesen (1975) distinguish into different basic emotions: anger, fear, enjoyment, sadness, surprise and disgust. The emotion anger demonstrates that there is no concrete emotion representing anger, but more than 60 different variations of it. Each of these 60 variations share different facial muscular patterns that assign them to the anger family instead of any other emotion family (Ekman and Friesen, 1975). These variations could occur due to differences in biology, learning experiences, or different conditions in which the emotion occurred. Regarding facial expressions, it is therefore very important to use standardized instruments for investigating emotional purposes to be able to control the evoked psychological and physiological responses (Goeleven et al., 2008).

A well-known database for facial expressions is the Radboud Faces Database (Langner et al., 2010). The Radboud facial database includes eight different facial expressions from adults, as well as children. The validation study included judgments on the expression itself, the intensity, clarity, genuineness of the expression and valence ratings. Results indicated that there was an 82% accordance between the intended and the finally chosen expression. However, it should be mentioned that faces representing surprise were occasionally confounded with fear or vice versa. Furthermore, expression effects were higher for happiness and lower for contempt compared to all other six emotions (neutral, anger, sadness, fear, disgust, and surprise). Taking the valence ratings into account, happiness was declared definitively positive, whereas neutral and surprise where categorized as neutral and all negative emotions where classified as such. Apart from the Radboud Faces database, the Karolinska Directed Emotional Faces (KDEF) database also exists and considers 490 frontal-colored-facial pictures form 70 individuals (35 female) representing seven different emotions (Goeleven et al., 2008). The KDEF database was used by Goeleven et al. (2008) to measure emotion, intensity and arousal, using the SAM-Scale (Bradley and Lang, 1994). Disgust showed the highest intensity ratings, which differed significantly from all other emotions, except happiness and surprise. The neutral emotional category achieved significant lower arousal ratings than all other emotions. A research study from Garrido and Prada (2017), using stimulus material from the KDEF database and collecting ratings for angry, neutral and happy faces on four different dimensions (attractiveness, familiarity, emotional intensity and valence), revealed that emotional stimuli compared to neutral ones achieved higher emotional intensity ratings. The valence scores were, as expected, most positive for happy faces, decreasing for neutral and angry stimuli (Garrido and Prada, 2017). Adolph and Alpers (2010) investigated valence and arousal ratings by taking into account two facial databases, the KDEF and the NimStim set (Tottenham et al., 2009). Their results indicate differences in valence ratings for different emotions, whereas angry achieved the lowest valence ratings and happy the highest; fearful, neutral, and surprise were listed in between the two. This emotion effect could also be observed for arousal values: Angry, happy and fearful faces revealed the same level of arousal, whereas surprise and neutral were less arousing than the before mentioned ones.

Some research has shown, that the emotion processing of stimuli can vary depending on valence and arousal (Bradley et al., 1992; Kuchinke et al., 2005). An investigation by Calvo and Lundqvist (2008) revealed faster reaction times for happy and neutral facial expressions (from the KDEF database) in contrast to fear, surprise, anger and sadness. Furthermore, they showed slowest reaction times for fearful expressions. The other discrete emotions, as mentioned before, ranged in between the two. Similar results have been found by Palermo and Coltheart (2004), collecting the reaction times for stimuli from five different facial databases and seven different discrete emotions, also indicating faster reaction times for happy stimuli than for all other discrete emotions and slowest for fearful stimuli. In contrast to these findings, research by Hansen and Hansen (1988) revealed shorter reaction times for finding an angry face within a happy crowd scenario, compared to finding a happy face in an angry-crowd scenario. Matching these results, an investigation from Eastwood et al. (2001) also found shorter reaction times for identifying the location of negative faces as well, compared to positive faces. Regarding the record of reaction times in the research field of emoji and emoticons, the main study from Kaye et al. (2021) should be mentioned, examining whether participants reacted faster to human faces or emoji. Their results showed, fastest reaction times for emoji compared to faces and words, whereas faces have been reacted to still faster than words. Furthermore, they did not find an interaction effect between stimulus and valence. An ERP study by Zhao et al. (2019) compared real face (taken from two facial databases) to cartoon faces, which could be seen as correspondent to emoji, investigating not only neural correlates, but also assessing reaction times. They could show reaction times where shorter for happy faces than for angry faces, but no significant results could be found by comparing real faces to cartoon faces. Moreover, results by Aldunate et al. (2018) suggested, that participants reacted faster to positive emoticons compared to negative ones. These results indicate that not the stimulus type plays a crucial role in terms of reaction times, but valence. For instance, in studies investigating affective judgments for emotional and neutral words, people often respond faster to positive self-related words than to self-related negative or self-related neutral words or negated control stimuli or when the same words are not self-related (Weis and Herbert, 2017; Meixner and Herbert, 2018). A positive valence effect of emotional processing could also be found within the word processing literature for subjective data (e.g. memory, Herbert et al., 2009), in affective word ratings irrespective of language (Dodds et al., 2015) and also in written as well as in spoken language in healthy adults (Augustine et al., 2011; Herbert et al., 2019). However, as far as emotional valence effects are concerned diverse results are available too. A study by Kousta et al. (2009) showed a preferred processing pattern for positive and negative stimuli, compared to neutral ones. Taken together, heterogeneous results have been found. Nevertheless, as aforementioned, a positivity-bias could be observed for the perception and processing of faces and emoticons. Whereas, no clear or significant effects have yet been reported, concerning the matter of interaction between emoji stimulus and emotion.

This leads to the last aim of this study, namely collecting reaction times of the answer time span of the participants in order to investigate whether differences between the stimulus categories and the discrete emotions, as well as a positivity bias, could be observed. To examine the aforementioned aims, the following three hypotheses were stated: (1) The emotionality ratings should not reveal any differences between faces and emoji, but both categories should differ significantly from emoticons. (2) Significant correlations between valence and arousal should be observed along the two axes, considering all stimulus categories and discrete emotions. (3) The fastest reaction times should be measured for happy faces, as they should be the best-known stimuli, followed by happy emoji and happy emoticons.

## Methods

### Participants

One hundred and thirteen participants took part in this study. However, some participants had to be excluded because of regular drug consumption (alcohol: *n* = 2, cannabis: *n* = 17, mixed drug consumption: *n* = 6) as well as mental diseases (*n* = 2) and drug consumption paired with mental diseases (*n* = 3). Mental, cardiovascular or neurological disorders as well as other drug consumption was assessed in a self-report amnestic interview. Afterwards, 83 participants (*n* = 74 female), aged between 18 and 49 years (*M* = 22.52, *SD* = 0.58) were included in the final data set. The majority of participants were German (96.4%), 79 participants indicated that German was their native language, but three of them were raised bilingually (1 × Polish, 2 × Turkish). However, four other participants indicated Russian (1 × ), Turkish (2 × ), and Greek (1 × ) as their native languages. Furthermore, 93.9% were students from the University of Ulm (Germany), 72 of them did not indicate which subject they are studying, three declared to study psychology and three others quoted to study and work in parallel. Furthermore, five participants stated to be non-students. Data acquisition was conducted via the online platform “Unipark” (Quest-Back, 2019). At the beginning of this online-based investigation, participants were asked to read and accept the participant information as well as the informed consent, stating the voluntariness of their participation. Also, data secrecy and storage issues were explained to and signed by the participants. Psychology students from Ulm University could be rewarded with credit points. This study was conducted with ethical approval from the local ethics committee from Ulm University, Germany (https://www.uni-ulm.de/einrichtungen/ethikkommission-der-universitaet-ulm/).

### Stimulus Material

The stimulus set used in this investigation included 60 stimuli: 18 emoji, 18 emoticons, and 24 faces representing six different emotional expressions. Facial stimuli were extracted from the Radboud database, whereas always two females and two male faces were included in one discrete emotion category (Langner et al., 2010). In total, eight different actors and eight different actresses were used to display the emotions. The emoji were used from Emojipedia, either in the representation of “iOS” or in the “What's App” version (for a list containing all used emoji, as labeled on Emojipedia, please see Supplementary Material). For each discrete emotion, three emoji have been assigned, likewise for emoticons. The emoticon stimuli were self-created with the software txt2bmp. Nevertheless, some inspiration was taken from the Emoticons Appendix.^1^ Furthermore, it should be mentioned that only western-style emoticons were used in this study. There were more facial stimuli than emoji and emoticons because the gender aspect had been considered. Hence, a balanced number of male and female faces were chosen. The selected stimuli were categorized in different discrete emotions, corresponding to Ekmans (1992) classification (happy, anger, fear, sadness, surprise) and a neutral condition. The disgust condition was left aside, because not enough representative emoji could be found for this category. The categorization of the stimuli to the discrete emotions was performed by two independent team members of the department of Applied Emotion and Motivation Psychology and the authors. Only stimuli (*n* = 60 as mentioned above), which have been congruently assigned to the same discrete emotion category by all raters, have been included in the investigation.

All stimuli were changed with Adobe Photoshop into black-and-white pictures, to avoid color effects on the affective ratings. Furthermore, the facial stimuli were edited that only the facial expression was visible. Because of contrast and brightness effects, emoji, and faces were edited with the IrfanView Version 4.42 software. The correction of the gamma values was between 1.0 and 1.5 for the faces and 1.3–1.7 for the emoji. Afterwards, all stimuli were adjusted in size, also with Adobe Photoshop Version 9.0. Emoji had a size of 280 × 295–290 × 300 pixel, faces of 270 × 380–300 × 380 pixels and emoticons of 120 × 120–120 × 135 pixels. The font size of the emoticons varied from 48 pt to 72 pt, regarding the length of the code. To avoid that some stimuli might appear very blurred, these were corrected with the GIMP Version 2.8 software on pixel level. The rating for each dimension of each stimulus was presented on a single page and the presentation duration was controlled by the participants.

### Procedure and Measures

**Figure 1:**
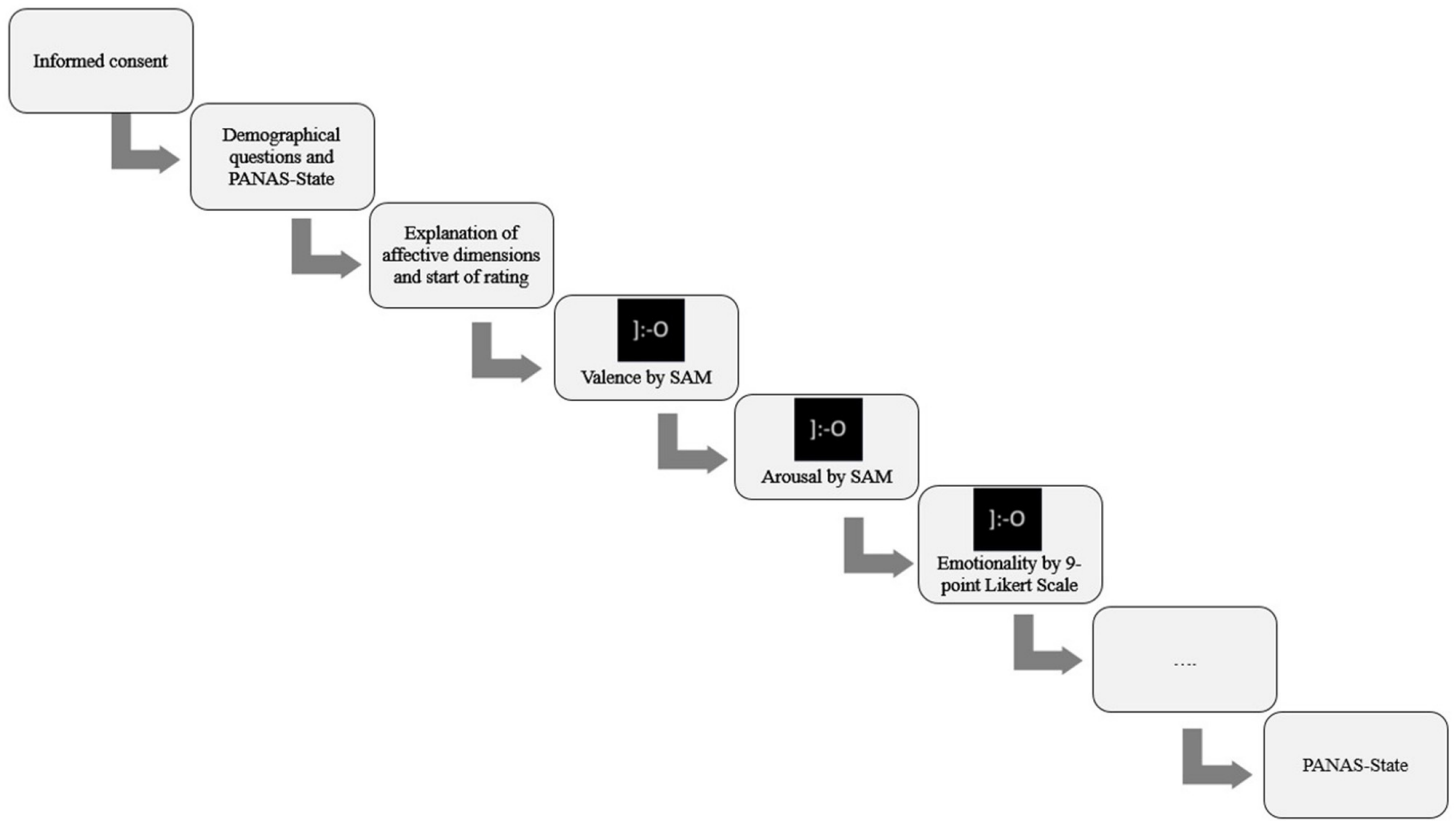
Schematic representation of the experimental procedure.

The online survey started with demographical questions (such as age, gender and nationality). To measure the momentary mood of the participants, the German Positive and Negative Affect Schedule (PANAS)—in the State version—was used (Janke and Glöckner-Rist, 2014). The PANAS consist of a 7-point-likert scale, measuring positive and negative affect and was used in this study before and after the affective rating of the stimuli. To state their answer regarding the affective ratings of the stimuli, the 9-point Self-Assessment-Manikin-Scales (SAM) for valence (from 1 = “unpleasant/negative” to 9 = “pleasant/positive”) and arousal (from 1 = “calm” to 9 = “arousing”) were used (Bradley and Lang, 1994). Finally, the emotional aspect of the stimuli should be rated. For this purpose, participants were instructed as followed: “Please indicate how intense the stimulus represents the emotion ‘*surprise*’.” Afterwards, they were able to give their opinion on a 9-point Likert scale, with 1 indicating “not at all,” five “partly,” and nine “totally.” The participants were forced to make a choice. Participants always rated valence first, followed by arousal and lastly emotionality. Each subjective rating was conducted on a separate page of the questionnaire. Stimuli were always presented in the same order, starting with a fearful emoji, followed by a fearful face (gender has been alternated for discrete emotions) and lastly a fearful emoticon. After fear, the discrete emotions were presented in this order: Happiness, neutral, sadness, surprise, and anger. This was done to prevent a negative influential bias concerning the ratings of the stimuli (for a schematic depiction of the procedure see Figure 1). Participants were instructed to answer as accurately and fast as possible, therefore no temporal deadline was set. However, after 1 h of no reaction, the survey did abort. When they made their choice for a stimulus, the next page with the next subjective rating was shown.

To complete the survey ~45 min were required. This study represents a quasi-experimental design, as there was no control group involved. The affective ratings (for valence, arousal, and emotionality) of the stimuli as well as the reaction times of the ratings were assessed as dependent variables. Concerning the reaction times, it should be mentioned that these cannot be compared to reaction times from a laboratory setting. It would be more appropriate to say that we assessed the response time participants needed to fulfill the rating of each stimulus. The three different stimuli types (faces, emoji and emoticons) as well as the different discrete emotions (anger, fear, sadness, happiness, surprise, and neutral) were assessed as independent variables.

### Data Analysis

The analysis of the data was performed with the software IBM SPSS Statistics 26.0.0.1 (IBM Cooperation, 2017). The subjective ratings of emotionality as well as the assessed reaction times were analyzed in separate two way repeated measurement analyses of variance (ANOVAs) using the factors stimulus categories (faces, emoji, and emoticons) and discrete emotions (fear, anger, sadness, happiness, surprise and neutral). Dependent T-tests were conducted *post-hoc* for further analysis. Before conducting the aforementioned analysis all statistical requirements have been reviewed and adapted if necessary. ANOVAs' results are reported with Greenhouse-Geisser corrections when needed. Significance values (*p*) of *post-hoc* tests were controlled for multiple comparisons according to the procedure suggested by Benjamini and Hochberg (1995).

Affective Ratings of valence and arousal were evaluated by Pearson's product-moment correlations, whereas *r* = 0.10 indicates a small correlation, *r* = 0.30 a medium and *r* = 0.50 a high correlation (Cohen, 1988). Also, it should be mentioned that the mood, assessed with the PANAS State, decreased significantly from pre to post measurement for the positive affect [respectively: *M* = 26.98, *SD* = 6.19, *M* = 24.53, *SD* = 6.46, *t*_(82)_ = 5.28, *p* < 0.01] as well as for the negative affect [respectively: *M* = 12.73, *SD* = 2.80, *M* = 12.16, *SD* = 2.62, *t*_(82)_ = 2.33, *p* < 0.05].

By subtracting the start time (when the page set open and the stimulus was presented) from the response time (when participants clicked and made their choice) reaction times were calculated. Reaction times within a time window of 0–300 s have been included into data analysis.

## Results

### Emotionality Ratings

**Figure 2:**
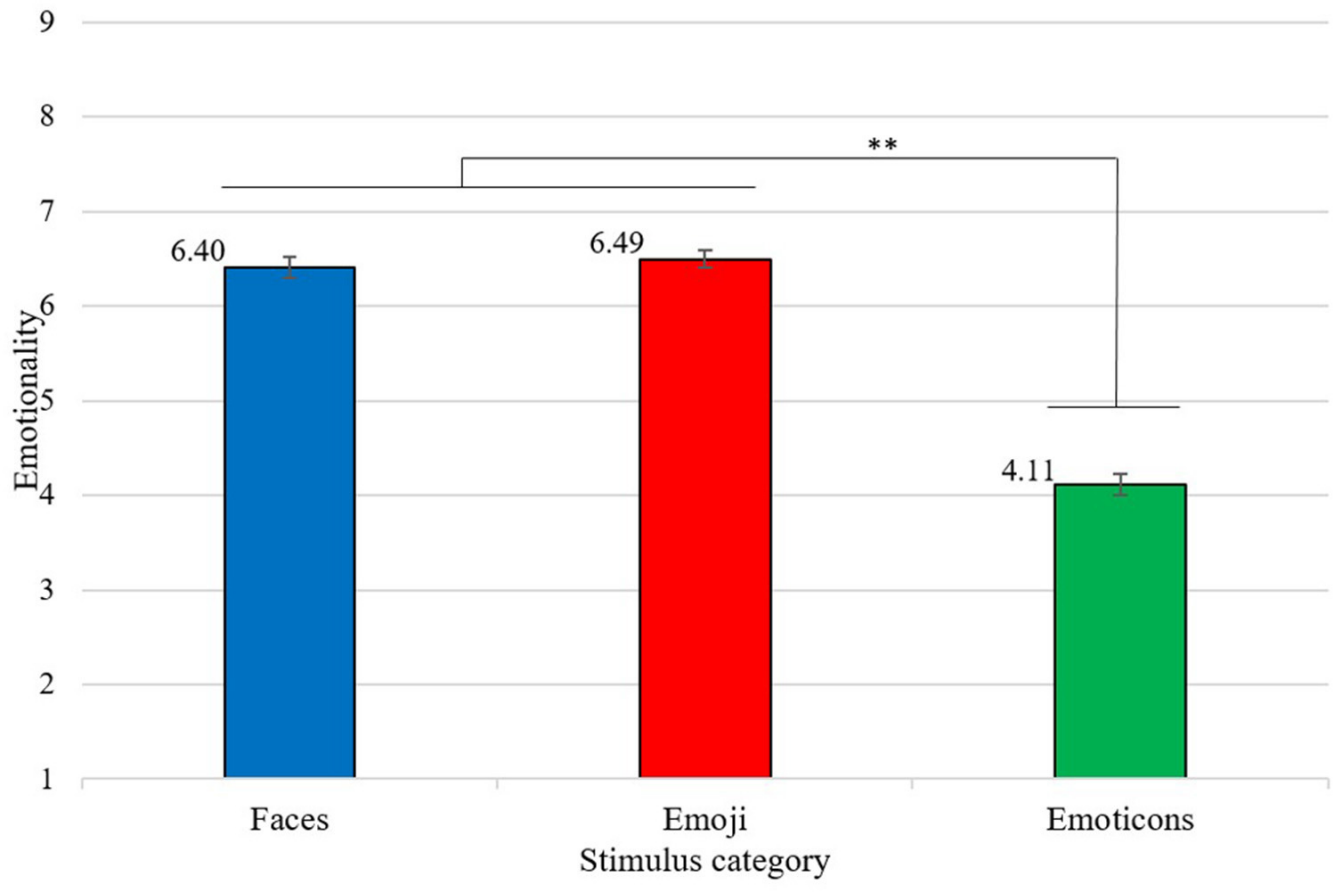
Mean emotionality scores for each stimulus category, from 1 = “not at all,” to 9 = “totally.” Error bars depicted in standard errors *(SE)*. **p < 0.01.

Descriptive analysis of the subjective ratings of emotionality showed that there were differences in emotionality values across stimulus categories as well as across discrete emotions. Regarding the stimulus categories, 95.8% of faces were rated with a score of 5 (“partly”) or higher, also emoji achieved 83.3% in this value range. In contrast, emoticons showed that 61.1% were rated five or below. Also, a significant medium and negative correlation between the emotionality ratings and the stimulus categories could be found (*r* = −0.55, *p* < 0.01). However, for the discrete emotions the results vary. Happiness and surprise revealed quite high percentages for ratings five or higher (100% and 90%, respectively). Fear and sadness were at 70% and anger as well as neutral at 60%. The results of the conducted two way repeated measures ANOVA revealed a significant main effect for stimulus category [*F*_(1.78,145.79)_ = 348.96, *p* < 0.01, partial η^2^ (η^2^p) = 0.81]. This effect represents significantly lower emotionality values for emoticons compared to emoji [Mean Difference = *MD* = −2.38, 95%-CI [−2.60, −2.16], *p* < 0.01] and human faces [*MD* = −2.29, 95%-CI [−2.58, −2.00], *p* < 0.01, see Figure 2].

Furthermore, a significant main effect of the discrete emotion occurred [*F*_(3.82,313.32)_ = 85.88, *p* < 0.01, η^2^p = 0.51]. Fear differed significantly from all other discrete emotions, except neutral, stating lower emotionality scores (see Table 1). Anger showed significantly weaker emotionality scores compared to happiness, surprise and neutral. This also holds true for sadness. In comparison, the discrete emotion of happiness differed significantly from surprise and neutral with more positive emotionality values. In addition, surprise also showed significantly more positive emotionality scores compared to neutral.

**Table 1:** Mean Differences of emotionality scores for all discrete emotions.

**Discrete emotions**		**MD**	**95%-CI**	**SE**	**p**
Fear	*Anger*	−1.05	[−1.40; −0.71]	0.11	<0.01^**^
	*Sadness*	−0.93	[−1.25;−0.61]	0.11	<0.01^**^
	*Happiness*	−2.19	[−2.56; −1.82]	0.12	<0.01^**^
	*Surprise*	−1.52	[−1.86; −1.19]	0.11	<0.01^**^
	*Neutral*	−0.36	[−0.80; 0.09]	0.15	0.26
Anger	*Sadness*	0.12	[−0.16; 0.41]	0.09	1
	*Happiness*	−1.14	[−1.44; −0.84]	0.10	<0.01^**^
	*Surprise*	−0.47	[−0.78; −0.16]	0.10	<0.01^**^
	*Neutral*	0.70	[0.22; 1.17]	0.16	<0.01^**^
Sadness	*Happiness*	−1.26	[−1.55; −0.97]	0.09	<0.01^**^
	*Surprise*	−0.59	[−0.91; −0.27]	0.11	<0.01^**^
	*Neutral*	0.57	[0.20; 0.94]	0.12	<0.01^**^
Happiness	*Surprise*	0.67	[0.34; 1.00]	0.11	<0.01^**^
	*Neutral*	1.84	[1.40; 2.27]	0.14	<0.01^**^
Surprise	*Neutral*	1.17	[0.71; 1.62]	0.15	<0.01^**^

*MD, Mean differences; CI, 95% confidence interval; SE, Standard error*;

** *p < 0.01*.

**Figure 3:**
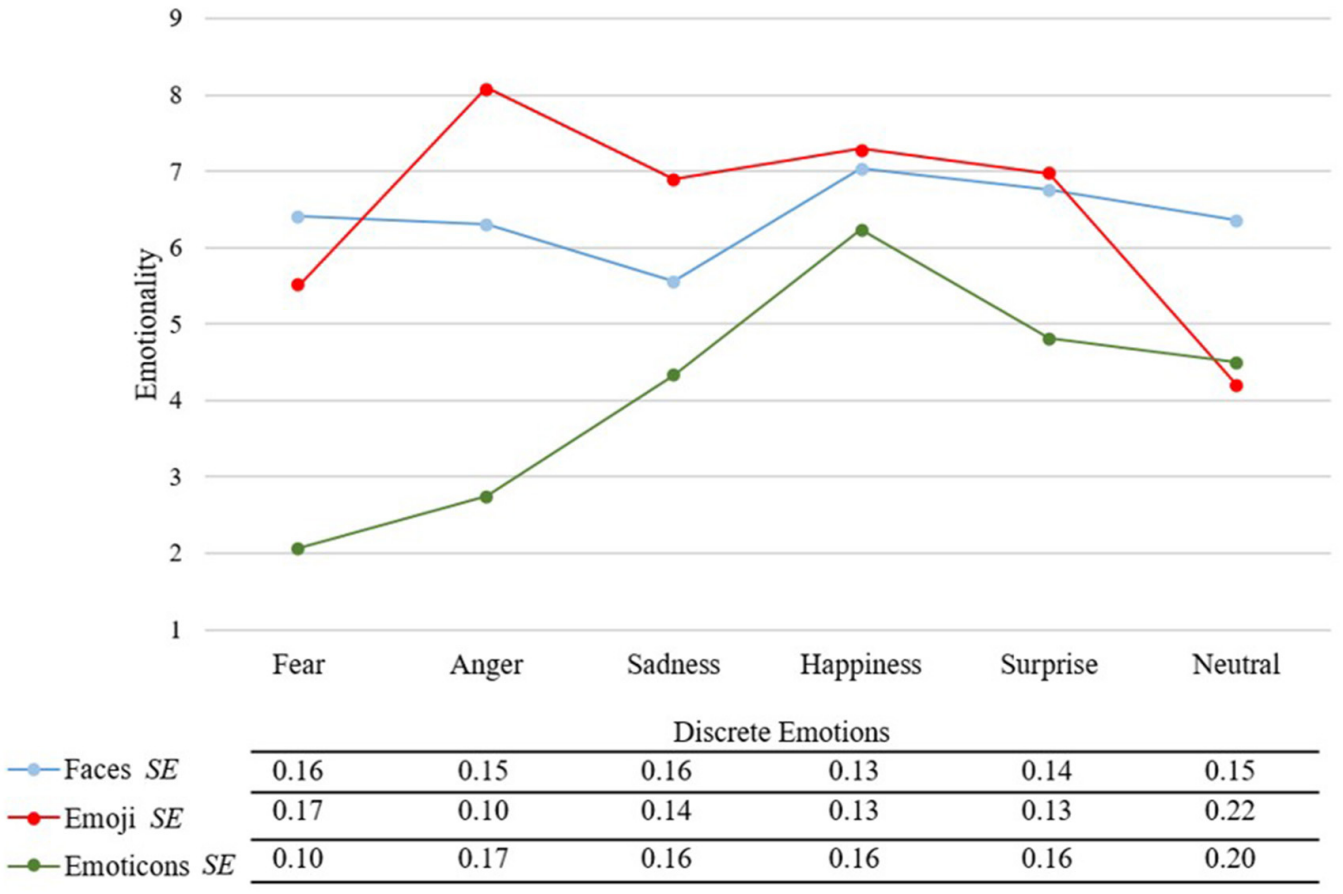
Mean emotionality scores for each stimulus category and discrete emotion, from 1 = “not at all” to 9 = “totally” and standard errors (*SE*).

Moreover, the interaction effect of stimulus category and discrete emotion became significant [*F*_(6.87,563.65)_ = 111.21, *p* < 0.01, η^2^p = 0.58, see Figure 3]. A *post-hoc* conducted paired *t*-test revealed that all stimulus categories differed significantly from each other for the discrete emotion of fear (see Table 2). Within the discrete emotion of fear, faces achieved highest emotionality values followed by emoji and then by emoticons. For the emotions anger and sadness also all stimulus categories differed significantly from each other (emoji > faces > emoticons). Happy faces and emoji did not differ significantly, but both categories differed significantly from happy emoticons. The same pattern of results could be found for the discrete emotion of surprise. Considering the neutral discrete emotion, faces did show significant differences compared to emoji and emoticons, whereas the latter two did not differ significantly.

**Table 2:** Results from *post-hoc* conducted *t*-test for emotionality for all discrete emotions paired with all stimulus categories.

		***M***	***SD***	***t***	***SE***	***p***
Fear	Faces vs. emoji	0.89	1.59	*t* (82) = 5.11	0.17	<0.01^**^
	Faces vs. emoticons	4.34	1.41	*t* (82) = 28.1	0.15	<0.01^**^
	Emoji vs. emoticons	3.45	1.50	*t* (82) = 20.99	0.16	<0.01^**^
Anger	Faces vs. emoji	−1.78	1.26	*t* (82) = −12.90	0.14	<0.01^**^
	Faces vs. emoticons	3.56	1.83	*t* (82) = 17.68	0.20	<0.01^**^
	Emoji vs. emoticons	5.34	1.67	*t* (82) = 29.21	0.18	<0.01^**^
Sadness	Faces vs. emoji	−1.34	1.63	*t* (82) = −7.46	0.18	<0.01^**^
	Faces vs. emoticons	1.22	1.71	*t* (82) = 6.51	0.19	<0.01^**^
	Emoji vs. emoticons	2.56	1.46	*t* (82) = 16.04	0.16	<0.01^**^
Happiness	Faces vs. emoji	−0.26	1.08	*t* (82) = −2.19	0.12	<0.05^*^
	Faces vs. emoticons	0.79	1.42	*t* (82) = 5.07	0.16	<0.01^**^
	Emoji vs. emoticons	1.05	1.18	*t* (82) = 8.07	0.13	<0.01^**^
Surprise	Faces vs. emoji	−0.21	1.33	*t* (82) = −1.46	0.15	=0.15
	Faces vs. emoticons	1.95	1.58	*t* (82) = 11.25	0.17	<0.01^**^
	Emoji vs. emoticons	2.16	1.48	*t* (82) = 13.36	0.16	<0.01^**^
Neutral	Faces vs. emoji	2.15	2.22	*t* (82) = 8.83	0.24	<0.01^**^
	Faces vs. emoticons	1.86	1.89	*t* (82) = 8.95	0.21	<0.01^**^
	Emoji vs. emoticons	−0.29	1.68	*t* (82) = −1.57	0.18	=0.12

*M, Mean, SD; Standard deviation; SE, Standard errors*;

* *p < 0.05*,

** *p < 0.01*.

### Affective Space

**Figure 4:**
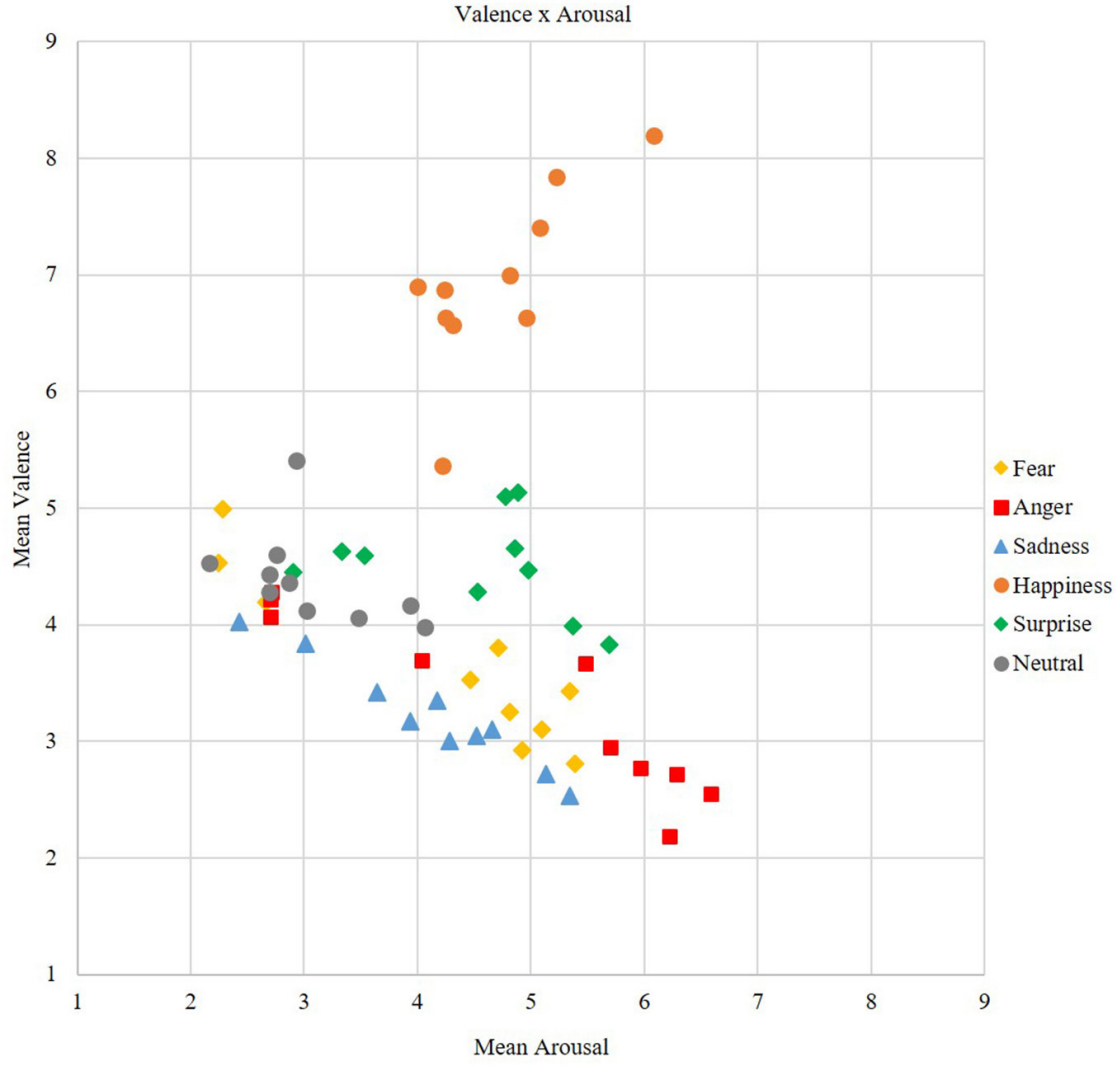
Mean corresponding valence and arousal scores for each discrete emotion.

**Figure 5:**
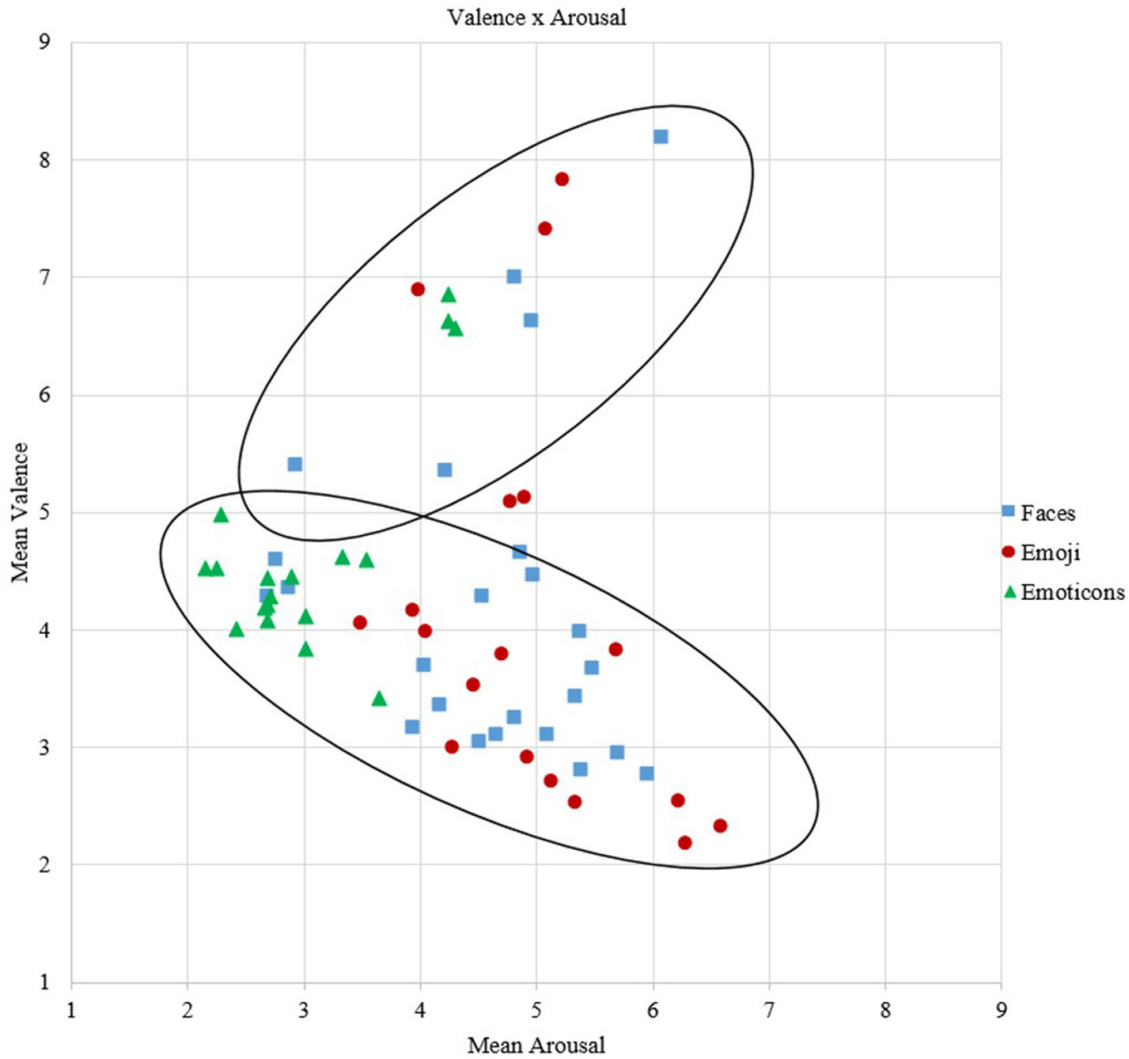
Mean corresponding valence and arousal scores for each stimulus according to the category.

Highest valence scores were achieved by happy stimuli (*M* = 6.94, *SD* = 0.90), surprise (*M* = 4.51, *SD* = 0.75), and neutral (*M* = 4.39, *SD* = 0.55) stimuli ranged in a neutral valence range and all negative discrete emotions were rated as negative [fear: *M* = 3.65, *SD* = 0.58; anger: *M* = 3.27, *SD* = 0.79; sadness: *M* = 3.22, *SD* = 0.77), see Figure 4]. In terms of stimulus categories, emoticons (*M* = 4.69, *SD* = 0.87) were rated more neutrally on valence compared to emoji (*M* = 4.11, *SD* = 0.56) and faces (*M* = 4.23, *SD* = 0.48), which were rated more emotionally (positive as well as negative, see Figure 5). Also, emoji (*M* = 4.95, *SD* = 1.14) revealed highest arousal values, followed by faces (*M* = 4.59, *SD* = 1.10) and lastly emoticons (*M* = 3.04, *SD* = 1.07). The discrete emotions of anger and happiness (*M* = 4.72, *SD* = 1.17) showed highest arousal values, followed by fear, sadness and surprise (respectively: *M* = 4.19, *SD* = 1.08; *M* = 4.11, *SD* = 1.16; *M* = 4.49, *SD* = 1.19), indicating a medium arousal level and the neutral (*M* =3.06, *SD* = 1.01) emotion with the lowest arousal scores (see Figure 5).

Valence and arousal scores showed a small, negative and non-significant correlation (*r* = −0.14, *p* = 0.30, *n* = 60). Splitting the valence scores at the crucial point of 5 in more negative and more positive valence ratings, the correlations with arousal became significant (*r* = −0.77, *p* < 0.01, *n* = 47; *r* = 0.56, *p* < 0.05, *n* = 13). Promoting these results, the unique boomerang-shape is recognizable in the data (see Figure 5).

Furthermore, the correlations of arousal and valence revealed significant results regarding the stimulus category. The correlation of valence scores below and above 5 and arousal scores for faces were significant, indicating a strong negative correlation and a strong positive correlation (see Table 3). Also, the correlation for lower valence values and arousal became significant for emoji, showing a high negative correlation.

**Table 3:** Correlations of valence and arousal for each stimulus category.

**Correlations Pearson‘s** ***r***	**Faces**	**Emoji**	**Emoticons**
Valence <5 × Arousal	*r* = −0.54^*^, *n =* 19	*r* = −0.78^**^, *n =* 13	*r* = −0.41, *n =* 15
Valence>5 × Arousal	*r* = 0.90^*^, *n =* 5	*r* = 0.16, *n =* 5	*r* = −0.75, *n =* 3

* *p < 0.05*,

** *p < 0.01*.

Moreover, the correlations for valence and arousal scores taking into account the discrete emotions have been investigated. The results show significant high correlations for the discrete emotion fear (*r* = −0.92, *p* < 0.01, *n* = 10), anger (*r* = −0.93, *p* < 0.01, *n* = 10), sadness (*r* = −0.97, *p* < 0.01, *n* = 10), and happiness (*r* = 0.76, *p* = 0.01, *n* = 10). No significant correlations have been found for surprise (*r* = −0.27, *p* = 0.44, *n* = 10) and neutral (*r* = −0.47, *p* = 0.18, *n* = 10).

### Reaction Times

**Figure 6:**
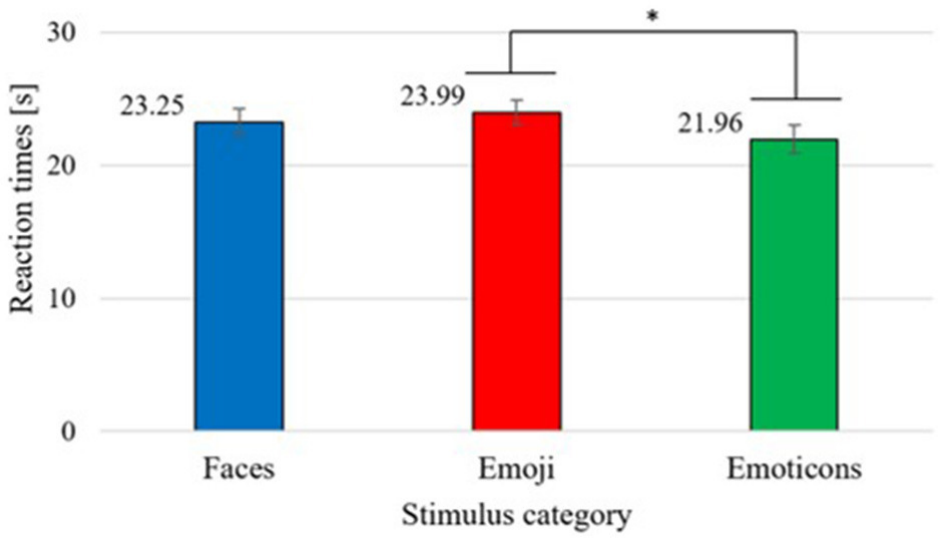
Mean reaction times in seconds for each stimulus category. Error bars depicted in standard errors (*SE*). *p < 0.05.

**Figure 7:**
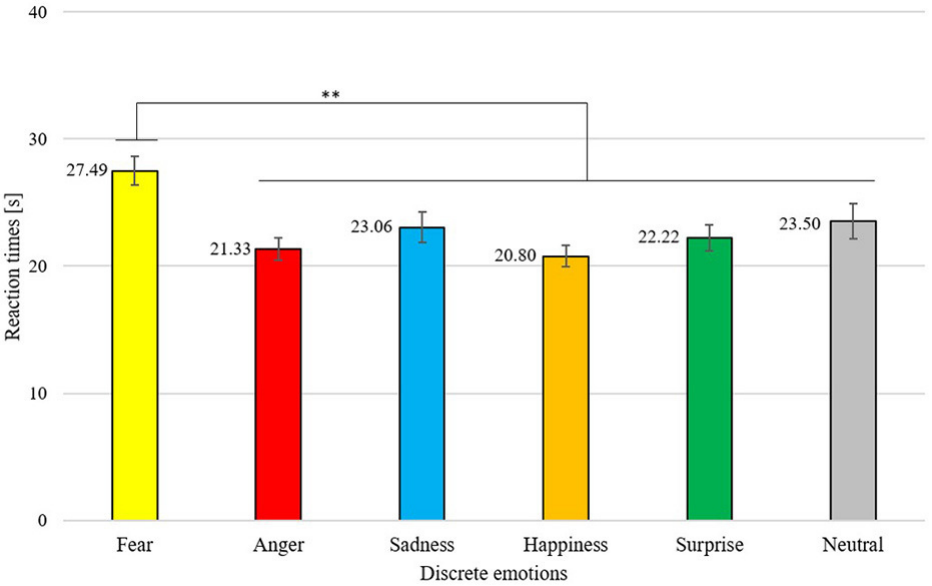
Mean reaction times in seconds for each discrete emotion. Error bars depicted in standard errors (*SE*). **p < 0.01.

Also, for testing the third hypotheses, a two way repeated measures ANOVA was conducted, revealing a significant main effect for the stimulus category [*F*_(1.79,146.66)_ = 3.73, *p* < 0.05, η^2^p = 0.04], showing significantly faster reaction times for emoticons compared to emoji [*MD* = −2.03, 95%-CI [0.02, 4.04], *p* < 0.05]. In a descriptive manner, it can be seen that emoticons achieved even faster reactions times than faces, however this effect did not become significant (see Figure 6). Also, the main effect for discrete emotions was significant [*F*_(3.59,294.35)_ = 12.55, *p* < 0.01, η^2^p = 0.13]. Only fear showed significantly larger reaction times compared to all other emotion dimensions (see Table 4). In Figure 7, it can be seen that participants reacted the fastest for happiness and anger, followed by surprise, sadness, neutral and lastly fear.

**Table 4:** Mean differences of reaction times for all discrete emotions.

**Fear**		***MD***	***SE***	**95% CI**	***p***
	Anger	6.16	0.85	[3.61, 8.72]	<0.01^**^
	Sadness	4.43	0.85	[1.86, 7.00]	<0.01^**^
	Happiness	6.69	0.66	[4.69, 8.69]	<0.01^**^
	Surprise	5.27	0.86	[2.66, 7.88]	<0.01^**^
	Neutral	3.99	1.09	[0.68, 7.29]	<0.01^**^

*MD, Mean difference; SE, Standard errors; CI, 95% confidence interval*;

** *p < 0.01*.

**Figure 8:**
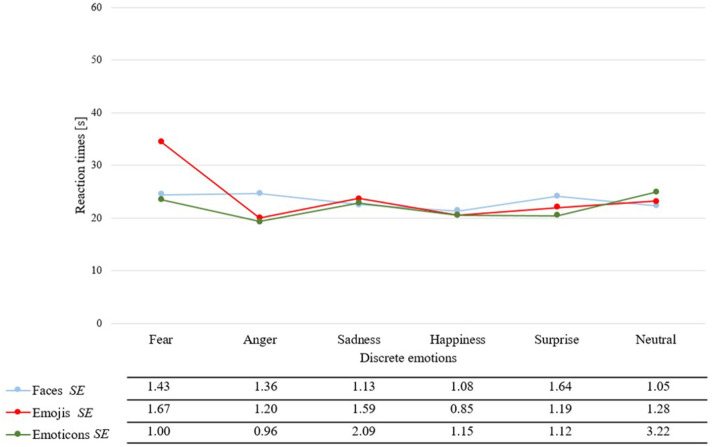
Mean reaction times and standard errors (*SE*) in seconds for each stimulus category paired with each discrete emotion.

Furthermore, the interaction effect of stimulus category and discrete emotions became significant [*F*_(4.86,398.49)_ = 5.93, *p* < 0.01, η^2^p = 0.07, see Figure 8]. A *post-hoc* conducted *t*-test showed, that the discrete emotion of fear revealed significant differences between faces and emoji as well as between emoticons and emoji, whereas both categories revealed significant faster reaction times than emoji [respectively: *t*_(82)_ = −6.79, *SE* = 1.47, *p* < 0.01; *t*_(82)_ = 6.90, *SE* = 1.58, *p* < 0.01]. Focusing on the discrete emotion of anger, emoji and emoticons did not distinguish significantly, but emoticons have been rated faster than faces [*t*_(82)_ = 4.02, *SE* = 1.32, *p* < 0.01] as well as emoji compared to faces (*t*_(82)_ = 2.86, *SE* = 1.61, *p* < 0.01). Concerning surprise there was a significant difference between emoticons and faces, whereas emoticons have been rated faster (*t*_(82)_ = 2.42, *SE* = 1.52, *p* < 0.01). All other discrete emotions revealed no statistical significant differences in reaction times over stimulus categories.

## Discussion

The first hypothesis stated no differences in emotionality scores between the stimulus categories of faces and emoji, but higher ratings for both faces and emoji compared to emoticons. Already within the descriptive data lower emotionality scores could be observed for emoticons compared to emoji and faces. Additionally, the discrete emotions of anger and neutral were rated lower compared to all other emotions. The conducted ANOVA revealed a significant main effect for stimulus category, representing significantly higher emotionality scores for faces and emoji compared to emoticons. However, no significant distinction between faces and emoji could be found. These results support the first hypothesis and suggest that faces and emoji represent emotional content similarly, whereas emoticons are not so clear. Rodrigues et al. (2018) also investigated the dimensions of clarity (“How clear or ambiguous is this stimulus”) as well as meaningfulness (“Please indicate to what extent this stimulus conveys a meaning/emotion”). They found that emoji were rated more plainly and more meaningful, in an overall perspective, compared to emoticons. Similar to our results, emoji achieved 79.08% on a high clarity level, whereas emoticons reached 50.59% on a low clarity level, indicating higher ambiguity (Rodrigues et al., 2018).

Moreover, the second significant main effect of discrete emotions showed highest emotionality values for the discrete emotion of happiness compared to all other stimulus categories. Happiness was followed by the discrete emotion of surprise, then neutral. Sadness and anger take the forth position and lastly fear achieved lowest emotionality scores. Garrido and Prada (2017) analyzed the dimension of emotion intensity for happy, angry, and neutral faces (on a seven-point Likert scale) which could be compared to the emotionality ratings that were collected in this study. Their results revealed higher emotional intensity ratings for happy and angry stimuli than for neutral stimuli. These findings partially contradict the results found here. Happiness was also rated highest, but was then followed by surprise and neutral. However, it needs to be mentioned that Garrido and colleagues investigated the KDEF database and took only three discrete emotions into account, whereas present data comprise emoji and emoticons and six discrete emotions. Besides, it should be mentioned that surprise as an emotion has quite a popular status, as this emotion can be positive (being surprised, because of getting a gift) as well as negative (being surprised, because of a thief in the house). This could of course be a possible explanation why the discrete emotion of surprise was categorized on a medium level of emotionality ratings. Also, neutral was rated as medium on emotionality which can have several reasons. First, the main effect comprises all types of stimulus categories, including emoticons, which might have pushed the mean into this medium area, because of unfamiliarity. Second, within the neutral category of emoji, symbols and facial emoji can be found which often obtain a negative connotation (Novak et al., 2015), as e.g., 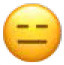
^2^, which looks a bit grumpy. Further neutral emoji or emoticons are rarely used in CMC, whereas it is normal to have a neutral facial expression. That is what makes it difficult to really take this dimension as a neutral comparison value.

In terms of interaction, a surprising effect was found: Not happiness as emotion achieved the highest emotionality ratings, as maybe expected, but angry emoji. These stimuli differed significantly to angry faces and angry emoticons. Also, the same result pattern could be found for sad emoji. These effects could be explained by pointing out results found by Jones et al. (2020), which showed, that negative and neutral emoji stimuli were rated more negatively by women than by men. As the majority of the participant sample underlying this study was female too, this is an important point to consider. Furthermore, it should be taken into account, that the findings by Jones and colleges are based on valence and not emotionality ratings. However, it should be mentioned too, that we did not find this effect for the negative discrete emotion of fear. Another possible explanation for the aforementioned effects, could be the genuineness of a human face (Langner et al., 2010). If the emotion is not evoked in a natural manner, it could be perceived as feigned. At this point, it should be remembered that faces from the Radboud Faces Database were used, where they also investigated the genuineness of a facial expression (Langner et al., 2010). Their results revealed that neutral and happy faces were rated as quite genuine, whereas all other emotions scored around the mean. This fits the present findings, indicating that maybe emotions as anger or sadness represented by human faces need to be evoked by a real, genuine stimulus and not only by an instruction. Yet, a reason for this result could be that the emotion of happiness or joy does not only include facial emoji, but also gestures, objects or other symbols (Novak et al., 2015; Shoeb and de Melo, 2020). Additionally, it should be mentioned that face emoji which are exaggerated, as e.g., 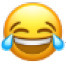
^2^ or 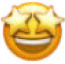
^2^, could play a crucial role in this emotion category. Within this study, a great effort to only investigate emoji with a high number of facial cues was made to allow a better comparison to human faces. Nevertheless, it should be mentioned as well, that happiness or joy is, as Ekman would classify it, an emotion family. Therefore, it exists of course a very broad range of emotional variations. That is why in further research we should also take emotional aspects as fun, love or humor into account. The smallest emotionality scores were shown by fearful emoticons. Nevertheless, the descriptive data showed that fearful stimuli represented the emotion up to 70%. Within the interaction effect, it became clear that this low emotionality value was due to the stimulus category of emoticons. Probably, as already said, because of the rare use and consequently the lack of familiarity, as well as unusual representations (e.g.,]:-O). In general, emoticons were rated lower than emoji and human faces, except for neutral. Moreover, happy emoji and faces did not differ significantly, but both categories differed from emoticons; the same was true for surprise.

The second hypothesis postulated the typical boomerang shape and significant correlations between the valence and arousal values along the positive as well as the negative axis of the affective space. The findings showed a significant medium positive correlation between positive valence scores and arousal. Corresponding to that, a significant high negative correlation could be observed for negative valence scores and arousal. These results support the second hypothesis and account for the visible boomerang shape. Furthermore, within the descriptive data it could be seen that emoji and human faces were rated more emotional (positively and negatively) on the valence scale compared to emoticons which were more neutrally categorized. Similar results have been found by Rodrigues et al. (2018), where emoji have been separated into positive and negative valence parts and emoticons have been divided into three parts (negative, positive, and neutral). Considering the arousal scale, emoji achieved highest values, followed by human faces, which were then followed by emoticons. Also, higher arousal scores for emoji than for emoticons observed by Rodrigues et al. (2018) fit to the present results. This effect turned up again by investigating the correlation between valence and arousal scores, dependent on the stimulus categories. Positive and negative valence scores correlated significantly with arousal scores on a high positive and a medium negative level, respectively. Additionally, emoji achieved a significantly high negative correlation of valence scores below five with arousal. Beyond that, happiness revealed highest valence rates, especially happy emoji. Whereas, the discrete emotions of surprise and neutral were rated in a neutral value range and all negative emotions were rated as such. Angry emoji therefore achieved the most negative valence score. The same pattern of results has been described by Langner et al. (2010), investigating human faces. Garrido and Prada (2017) mentioned similar results as well. Stimuli representing the discrete emotions of anger and happiness scored highest on arousal, especially angry emoji. Fear, sadness and surprise were rated on a medium arousal level, whereas the neutral emotional condition showed the smallest arousal level. Therefore, neutral emoticons represented the lowest arousal value. These results reflect the findings from Goeleven et al. (2008), who took only faces into account. The correlations between valence and arousal for the discrete emotions of fear, anger and sadness were significant, negative and high. In contrast, happiness achieved a significant high correlation, but in a positive direction.

The third hypothesis claimed fastest reaction times for happy faces, followed by happy emoji and happy emoticons, as these stimuli should be the best known due to regular usage. This hypothesis also took into account the aspect of the positivity bias. However, the descriptive results showed that the reaction times for all three stimulus categories were quite close to each other, though emoticons were reacted to the fastest. The conducted two-way ANOVA showed a significant main effect of stimulus category, no significant differences between faces and emoji as well as between faces and emoticons. Indeed, a significant distinction between emoji and emoticons could be observed. At this point, it should be mentioned, that the collected reaction times are the answer time of the participants and are therefore not completely comparable to reaction times assessed in a laboratory setting. An investigation from Kaye et al. (2021) recorded reaction times in a lexical decision task, taking into account human faces, emoji and words, in a positive, negative and neutral valence manner. Their results showed significant faster reaction times for emoji compared to faces and words. However, no significant valence effect or interaction effect could be found within their data. Taken together, these results are not in line with the findings within this study. However, it should be considered as well, that making a binary decision (“yes/no”) is quite different from deciding on a nine-point scale, which probably would take more time. Nevertheless, a possible explanation for the effect found here could be that emoticons lack familiarity compared to emoji (Rodrigues et al., 2018). Additionally, the results within the first hypothesis also showed significantly lower emotionality scores for emoticons, which could suggest less intense emotion representations. Those could be followed by a faster reaction due to a lack of knowledge or uncertainty. An investigation from Britton et al. (2006) compared reaction times of faces to IAPS pictures. Their results showed faster reaction times for faces compared to the IAPS pictures. Comparing these results to the current findings, it could be argued that faces and emoji are quite similar to each other, therefore no differences in reaction times could be seen. Whereas, emoticons, consisting of ASC-II character sequences, belong to another sort of stimuli category and therefore reveal faster reaction times. Regarding the second main effect of discrete emotions, significantly lower reaction times for fearful stimuli have been observed compared to all other discrete emotions, which did not differ significantly from each other. However, happiness and anger achieved fastest reaction times. Similar literature investigating faces partly supports these findings, showing that fear revealed the highest reaction times whereas happiness showed the fastest (Calder et al., 2000; Leppänen et al., 2003; Calvo and Lundqvist, 2008). The differences in present results, namely that anger also achieved fast reaction times, could be due to the fact that not only faces but also emoji and emoticons were considered. A research investigation from Herbert and Sütterlin (2011) showed faster reaction times for emotional nouns in general compared to neutral nouns. Which partly explains current results because surprise and neutral as discrete emotions were rated rather neutral. However, in this case the discrete emotion of sadness should have shown faster reaction times as well, which was not the case. A significant interaction effect finally showed that in general there was no difference for all discrete emotions between faces, emoji and emoticons, except from fear and anger. Regarding anger, there was a significant difference between emoticons compared to faces. However, fearful emoji achieved highest reaction times, whereas angry emoticons were rated fastest. It could be assumed that not the stimulus category plays the crucial role, which would fit the results from the first main effect, but apparently the discrete emotion. Since reaction times to the discrete emotion of anger were very fast too, it could be argued that these stimuli were reacted faster to because of the Fight-or-Flight response (LeDoux and Phelps, 1993) and therefore allowed preferred processing of the stimuli. Buodo et al. (2002) stated that the affective value of a threating stimuli can influence the extent of attentional resources, maybe to be able to accomplish fast adaptations to the situation. However, their results showed differences in reaction times of attention resources 1s after stimulus presentation. Pointing out that threating stimuli needed less attentional capabilities compared to neutral or pleasant stimuli. However, within our results, fearful stimuli revealed highest reaction times, even though these stimuli could also activate the Fight-or-Flight response. But maybe, fearful stimuli have not been perceived as such a threat as angry stimuli, because they were not so intense and therefore participants needed more time to detect whether it is just a negative stimulus or a threatening stimulus. It is very interesting, though, that happiness revealed highest reaction times within the second main effect, whereas in terms of interaction this did not hold true any longer. This could indicate that the suspected positivity bias is superseded from threating stimuli.

As already mentioned, it is therefore quite important that future research investigates the reaction times for quite different stimulus categories as well as with various participant groups. It could be possible to find different effects in terms of investigating different generations. For example, a younger population with a higher smartphone use, growing up with emoji and learning them intuitively, also called *digital natives generation*, could show significant differences compared to an older aged sample of participants. Furthermore, cultural as well as gender differences, which could not be investigated within this study due to too few male participants, could especially influence the valence and arousal ratings, which have been shown, concerning the valence ratings by Jones et al. (2020). Female individuals rated negative and neutral emoji more negative in valence than men, but there was no gender difference in positive emoji (Jones et al., 2020). Whereas, it should be mentioned that next to the horizontal emoticons, which were considered here, also vertical emoticons, which are more often used in eastern cultures, should be investigated as well. Also Lu et al. (2016) described significant different patterns of emoji usage across countries, whereas emoji as stimuli are universal and can overcome language barriers. Moreover, Ljubešić and Fišer (2016) described that Asia reveals most tweets with emoji, followed by South America and Europe.

A quite new stimulus category could be the memoji, which are personally generated emoji, available on the smartphone. This category is able to fill the gap between picturesque emoji and human faces. It would be quite interesting to investigate differences in perception and emotion recognition between these two stimulus categories. Within this research, black and white stimuli were used to ensure an adequate comparison between the stimuli. Nevertheless, further research should also take the colors of emoji into account, because they can play an essential role in terms of emotion intensity. However, another important point to mention, is the objective categorization of the stimuli to the discrete emotion categories. Within future research, this should be done, by a highly varying independent group of individuals to avoid subjective influences in terms of classification.

The most important limitation to mention is the neutral emotion condition. As explained in Wieser and Brosch (2012), neutral stimuli are often getting influenced by the preceding emotion or other contextual cues, especially when no facial expression should be shown, as in the neutral category. Therefore, it was a matter of concern for us, to keep a fixed order of the discrete emotions of the presented stimuli to be able to prevent for negative influences on the neutral condition. Consequently, neutral stimuli have always been presented after happiness. Moreover, Lee et al. (2008) investigated whether neutral faces are classified as such, using an implicit measurement task. Their results showed that responses to neutral faces were more likely to those of negative faces as compared to positive faces. Huang et al. (2008) classified this emoticon :-| as uncertain while, in the data on hand, it was part of the neutral emoticon condition. That is why it is so important to make sure that neutral stimuli are not always following negative stimuli to obtain a correct comparison condition. For example, by considering plants or animal emoji as a neutral condition, as well as corresponding pictures for human faces and non-meaningful ASC-II character sequences for emoticons. Lastly, more affective dimensions could be taken into account, as for example done by Rodrigues et al. (2018).

To summarize, due to the novel approach that was used to compare emoji, human faces and emoticons, the current data indicates that emoji and faces are rated quite high on emotionality compared to emoticons. Thus, it could be argued that emoji and faces are quite good in representing the associated emotions and therefore also in reducing ambiguity. For certain emotions, emoji are even better than faces. In contrast, emoticons are unclear and ambiguous, even for the well-known discrete emotion of happiness. Moreover, these findings expand the already existing literature concerning the affective space and the boomerang shape, due to significant correlations between valence and arousal for emoji. Lastly, the described reaction times state that emoji were reacted to at least as fast as faces, for nearly all discrete emotions, except anger and fear.

## Data Availability Statement

The raw data supporting the conclusions of this article will be made available by the authors, without undue reservation.

## Ethics Statement

The studies involving human participants were reviewed and approved by Local ethics committee of Ulm University, Ulm, Germany. The participants provided their written informed consent to participate in this study.

## Author Contributions

BF drafted and wrote the manuscript. CH revised the manuscript for intellectual and scientific content. BF and CH conceptualized the study. BF designed the survey under supervision of CH. Data recruiting was conducted by BF. BF performed the data pre-processing, the statistical data analysis and data interpretation under supervision of CH. Tables and Figures were created by BF. All authors contributed to the article and approved the submitted version.

## Conflict of Interest

The authors declare that the research was conducted in the absence of any commercial or financial relationships that could be construed as a potential conflict of interest.

## Footnotes

^1^Available online at: https://en.wiktionary.org/wiki/Appendix:Emoticons.

^2^Available online at: https://emojipedia.org/.
