# Supplementary material for: Emoji as Affective Symbols: Affective Judgments of Emoji, Emoticons, and Human Faces Varying in Emotional Content
Source: Front Psychol. 2021 Apr 20;12:645173. doi: 10.3389/fpsyg.2021.645173 (PMC8093811; doi:10.3389/fpsyg.2021.645173)
Supplement: Supplementary file 1 [file Table_1.docx]

Supplementary Material

# Supplementary Table

Table 1 – Supplementary Material

*Emoji as named on Emojipedia (Emojipedia®, 2021) and corresponding to the used version.*

|  | Apple Version | WhatsApp Version |
| --- | --- | --- |
| Fear | anguished_face  frowning_face_with_open_mouth | anguished_face |
| Anger | angry_face | angry_face  pouting_face |
| Sadness | pensive_face  slightly_frowning_face | worried_face |
| Happiness | grinning_face | slightly_smiling_face  smile_face |
| Surprise | hushed_face  flushed_face | hushed_face |
| Neutral | neutral_face | neutral_face  expressionless_face |
